# Prognostic Values of Fluctuations in Serum Levels of Alanine Transaminase in Inactive Carrier State of HBV Infection

**DOI:** 10.5812/hepatmon.17537

**Published:** 2014-05-01

**Authors:** Hossein Farzi, Nasser Ebrahimi Daryani, Leila Mehrnoush, Shima Salimi, Seyed Moayed Alavian

**Affiliations:** 1 Department of Gastroenterology and Hepatology, Tehran University of Medical Sciences, Tehran, IR Iran; 2 Middle East Liver Diseases Center (MELD), Tehran, IR Iran

**Keywords:** Hepatitis B Virus, Alanine Transaminase, Hepatitis B e Antigen, Hepatitis B, Chronic

## Abstract

**Background:**

Current guidelines introduce periodic monitoring of serum alanine transaminase (ALT) as the first-line modality in follow-up patients, with a hepatitis B virus (HBV) inactive carrier state.

**Objectives:**

This study aimed to determine the incidence rate and patterns of ALT fluctuations and prognostic values for the development of chronic HBV e antigen (HBeAg)-negative hepatitis B (CHB), HBV surface antigen (HBsAg) seroclearance, and liver-related complications.

**Patients and Methods:**

Treatment-naïve patients with a chronic HBV infection, HBeAg(-)/HBeAb(+), normal ALT levels, and HBV DNA < 2000 IU/mL, were followed-up every 6-12 months by assessing serum ALT levels. Serum HBV DNA was measured in cases of elevated ALT levels.

**Results:**

A total of 399 patients were followed-up for 8.9 years; ALT > upper limit of normal (ULN, i.e. 40 IU/L) was detected in 103 (25.8%) patients, with an annual incidence rate of 2.9%. ALT elevation was associated with; male gender, age, and higher serum ALT levels at study entry. Among the cases of ALT elevations, 16 (15.5%) patients had ALT levels > 2 × ULN. There were 38 (36.9%) patients who had ALT levels that remained > ULN over six months, and 21 (20.4%) patients experienced at least two episodes of ALT elevations. In 15 (14.6%) patients, elevated ALT levels were associated with increased HBV replication (i.e. HBV DNA > 2 000 IU/mL) and these were considered as CHB. However, elevation of ALT levels, even in the absence of HBV replication, increased the risk for the development of CHB up to 8-fold in prospective follow-ups. HBsAg seroclearance, cirrhosis, and hepatocellular carcinoma were detected in 43 (10.8%), 4 (1%), and 1 (0.25%) patients, respectively.

**Conclusions:**

Fluctuations in serum ALT levels may change the prognosis of a HBV inactive carrier state.

## 1. Background

Hepatitis B virus (HBV) infection is one of the major health challenges worldwide, and it is estimated to affect more than 350 million chronic carriers globally (1). Chronic infection with the virus may cause a wide spectrum of outcomes and complications that vary from an inactive carrier state to active chronic hepatitis, and from normal liver function to cirrhosis, hepatocellular carcinoma (HCC), and liver-related death (2).

The inactive carrier state, is the most prevalent subtype of chronic HBV infection (3), and it is mainly defined by the absence of the hepatitis B e antigen (HBeAg), presence of the hepatitis B e antibody (HBeAb), low levels of viremia (i.e. HBV DNA < 2 000 IU/mL), and persistently normal levels of alanine transaminase (ALT) in the serum (4, 5). Patients in an inactive carrier state, in comparison with patients with chronic HBV hepatitis, are considered to show a benign course of the disease with; minor histopathologic damage to liver tissue (6-9), and a lower incidence of cirrhosis (10), or HCC (11).

However, several studies have reported that patients in an inactive carrier state, compared to normal healthy control populations, may be at higher risk for developing liver histopathologic changes (12), HCC, and liver-related death (1). Moreover, it has frequently been reported that patients with an inactive carrier state may experience reactivation of the infection into chronic HBeAg negative hepatitis, with an annual incidence rate of 1 to 3 per 100 patients (13). Although the natural history of patients with an inactive carrier state is relatively well characterized, the incidence of complications previously described above has led to questions with regard to the identification of prognostic risk factors in these patients. It has been reported that advanced age at study entry (14, 15), male gender (14-16), HBV DNA ≥ 1 000 IU/mL at study entry (16), HBsAg level ≥ 1 000 IU/mL at study entry (17), and genotype B (16), or C (18), of the virus may increase the risk for reactivation of the infection into chronic HBeAg negative hepatitis. Currently, chronic HBeAg negative hepatitis is characterized by periodic reactivation of the infection and active hepatitis, which is associated with elevated levels of liver aminotransferases and HBV DNA (4). Overall, according to the benign natural history of patients with an inactive carrier state, current guidelines such as the American Association for the Study of Liver Diseases (AASLD) and the European Association for the Study of the Liver (EASL), suggest that these patients may not need immediate antiviral therapy, and advice for periodic follow-ups and frequent monitoring of serum ALT levels is sufficient (4, 5).

## 2. Objectives

In this study, we aimed to evaluate the prevalence and patterns of ALT fluctuations over periodic follow-ups of patients with an inactive HBV carrier state. We also investigated the probable associations between enzyme fluctuations and disease outcomes.

## 3. Patients and Methods

### 3.1. Study Design and Patient Population

This study took place from 2000 to 2013, and it was designed to follow-up patients with chronic HBV infections (i.e. HBsAg positive > 6 months) who were in an inactive carrier state and registered at the Tehran Hepatitis Center (THC: the outpatient clinic of the Baqiyatallah Research Center for Gastroenterology and Liver Diseases, Tehran, Iran). Inactive carrier state was defined as; HBeAg negative, HBeAb positive, HBV DNA < 2 000 IU/mL at study entry, and persistently normal levels of ALT through the first year of follow-up. Initial assessment of the patients included; detailed medical history and physical examination, familial history of liver disease or HCC, complete blood count with platelets, prothrombin time (PT), serum albumin, aspartate transaminase (AST), alanine aminotransferase (ALT), alkaline phosphatase (ALP), HBeAg/anti-HBe, HBV DNA, and abdominal ultrasonography. Patients with any of the following criteria were excluded from the study: history of receiving anti-HBV therapy; regular alcohol consumption; immunosuppressed patients (receiving immunosuppressive drugs, end stage renal disease and hemodialysis); thalassemia major; coinfection with HCV, HDV or HIV; concurrent liver diseases (cirrhosis, Wilson’s disease, autoimmune hepatitis, α-1-antitrypsin deficiency, biopsy proven non-alcoholic fatty liver disease or steatohepatitis) and abnormal findings through laboratory tests and ultrasonography at study entry. Informed consent was obtained from all participants in the study; and study protocols conformed to the ethical guidelines of the 1975 Declaration of Helsinki.

Patients were followed up every 6 to 12 months by assessing serum ALT levels; and annually with HBV serological markers (HBsAg, HBeAg). In cases of ALT > upper limit of normal (ULN, i.e. > 40 IU/L), serum HBV DNA was measured. Maximal serum ALT levels, frequency of ALT elevations and duration of each episode of ALT elevation were recorded. Drug induced hepatotoxicity or super-infection of HCV, HDV or HIV were evaluated in suspicious cases. Levels of liver aminotransferases were evaluated using standard ELISA kits (ULN = 40 IU/L). Serum HBV DNA levels were assessed by quantitative polymerase chain reaction assay (Cobas Amplicor HBV Monitor, Roche Diagnostics, sensitivity 200 copies/mL, conversion factor = 5.26).

### 3.2. Study Endpoints

Patients with ALT > ULN and HBV DNA > 2 000 IU/ml; whether HBeAg negative or positive, were considered as chronic hepatitis B (CHB). The decision to initiate anti-HBV therapy was made upon clinical evaluation, liver biopsy findings, or vibration-controlled transient elastography test (FibroScan 502 Touch, Echosens, USA). HBsAg seroclearance was defined as negative results for serum HBsAg on two consecutive occasions at least six months apart, which were maintained to the end of the study, with undetectable serum HBV DNA, and normal ALT levels. HBsAg seroclearance with development of HBsAb was considered as HBsAg seroconversion. Screening and diagnosis of cirrhosis and HCC were performed according to the previously described protocol (19).

### 3.3. Statistical Analysis

Quantitative variables were described as mean ± standard deviation (SD). Independent and paired-samples t-test, chi-square, and linear and binary logistic regression tests, were applied in cases of relevance. Descriptive and analytic statistics were performed using SPSS software version 19 (IBM Corporation; NY; USA). Differences or correlations (Pearson’s or Spearman’s) with P < 0.05 were considered statistically significant.

## 4. Results

### 4.1. Baseline Characteristics

Initially, 447 patients met our inclusion criteria. Considering exclusion criteria; 399 inactive hepatitis B carriers with normal serum ALT levels and HBV DNA < 2 000 IU/mL, were included in the study; 231 (57.9%) males and 168 (42.1%) females. Mean age for the patients diagnosed with a HBV chronic infection and at study entrance were 33.55 ± 11.64 and 35.57 ± 11.56 years, respectively. Patients were followed up for a mean period of 107.0 ± 32.8 months; ie, 8.9 ± 2.7 years. The period of follow-up in male patients was longer than for the females; 111.6 ± 36.2 vs. 100.5 ± 26.3 months (P < 0.001). Serum levels of AST, ALT, and ALP at study entry were 24.4 ± 7.8, 24.0 ± 9.4, and 171.3 ± 96.9 IU/l, respectively. No statistically significant association was found between age or HBV DNA at study entry, and serum levels of AST (P = 0.92, 0.66, respectively) or ALT (P = 0.16, 0.21, respectively). However, compared to females, male patients had higher levels of serum AST (26.0 ± 7.9 vs. 22.4 ± 7.0, P < 0.001) and ALT levels (26.9 ± 9.3 vs. 20.0 ± 7.9 IU/L, P < 0.001).

At study entry, 166 (41.6%) patients had undetectable serum HBV DNA. Mean serum viral load in 233 (58.4%) patients with detectable HBV DNA was 492.1 ± 559.4 IU/mL. No significant statistical correlation was found between; gender, age, AST, ALT or ALP levels at study entry, and detectable/undetectable HBV DNA, or quantitative PCR Hepatitis B viral load (P > 0.05).

### 4.2. Natural Course of ALT Fluctuations

**Figure 1.**
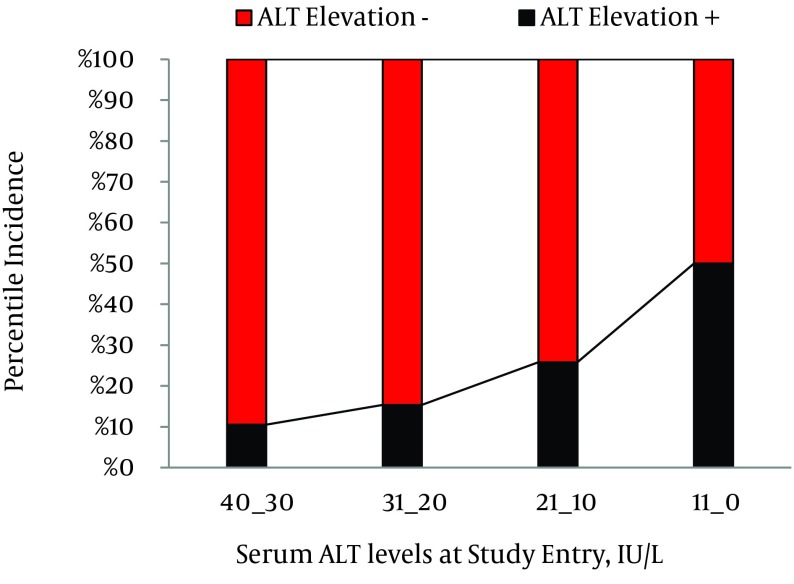
Association Between Incidence of Serum ALT Elevations and ALT levels at Study Entry (IU/L)

Through 3 557 person-years of follow-up, 296 (74.2%) patients had persistently normal levels of serum ALT, while 103 (25.8%) patients experienced at least one episode of ALT > ULN with an annual incidence rate of 2.89%. Among patients with ALT elevation, in 87 (84.5%), 10 (9.7%), and 6 (5.8%) persons, maximal serum ALT levels were within the range of 1-2 × ULN, 2-3 × ULN, and 3-4 × ULN, respectively. In 65 (63.1%) patients, ALT levels decreased to normal levels within six months; while in 38 (36.9%) patients, ALT levels remained over ULN for at least six months. Furthermore, 21 (20.4%) patients experienced at least two episodes of ALT elevations. The association between patterns of ALT fluctuations and probable underlying risk factors were evaluated and demonstrated in Table 1. Briefly, elevation of ALT > ULN had a significant statistical association with male gender (P < 0.001, RR = 2.7, 95% confidence interval [CI] = 1.8-4.1), lower age at study entry (33.4 ± 10.0 vs. 36.3 ± 11.9 years, P = 0.015), and higher serum ALT levels at study entry (28.8 ± 10.5 vs. 22.3 ± 8.3 IU/L, P < 0.001). In patients with ALT ≤ 10 IU/L, the cumulative incidence of enzyme elevation was 10.5%; compared to 50.0% in patients with ALT ≥30 IU/l at study entry (Figure 1). Mean level of serum ALT at the end of study was 26.33 ± 15.8 IU/L; ranging from 4 to 140 IU/L. ALT levels were significantly higher in the male patients (30.9 ± 18.2 vs. 20.0 ± 8.3 IU/l, P < 0.001).

A paired-samples t-test also revealed mild elevation in ALT mean levels in comparison to ALT measures at study entry (26.5 ± 16.0 vs. 24.0 ± 9.4 IU/L, P < 0.001). No significant association was found between; terminal levels of ALT levels and age at study entry, age at study ending, or HBV DNA measures at study entry (P = 0.09, 0.17, and 0.15, respectively).

**Table 1.** Patterns of Serum ALT Fluctuations in Inactive HBV Carrier State ^a^, ^b^, ^c^

	Persistently Normal ALT Levels	Maximal Serum ALT Levels			Presence of ALT > 6 Months	≥ 2 Episodes of ALT Elevations
		> ULN	> 2 × ULN	> 3 × ULN		
**Total number**	296	103	16	6	38	21
**Gender**						
Male	150 (50.7)	81 (78.6)	10 (62.5)	5 (83.3)	33 (86.8)	18 (85.7)
Female	146 (49.3)	22 (21.4)	6 (37.5)	1 (16.7)	5 (13.2)	3 (14.3)
P value		< 0.001 ^d^	0.36	0.21	< 0.001 ^d^	0.002 ^e^
**Family history of HBV infection**						
Positive	141 of 173 (81.5)	39 of 53 (73.6)	8 of 10 (80)	3 of 3 (100)	17 of 21 (55.3)	8 of 11 (72.7)
Negative	32 of 173 (18.5)	14 of 53 (26.4)	2 of 10 (20)	-	4 of 21 (19)	3 of 11 (27.3)
P-value	-	0.21	0.9	0.41	0.95	0.47
**Age at study entry, y**	36.3 ± 12.0	33.4 ± 10.0	31.2 ± 10.4	35.8 ± 8.4	31.9 ± 8.8	29.4 ± 9.0
P-value	-	0.018 ^f^	0.09	0.91	0.008 ^e^	0.003 ^e^
**Serum AST at study entry, IU/L**	23.9 ± 7.9	25.9 ± 7.2	24.8 ± 5.4	25.5 ± 4.5	26.3 ± 7.4	27.2 ± 8.0
P-value	-	0.026 f	0.68	0.62	0.08	0.068
**Serum ALT at study entry, IU/L**	22.3 ± 8.3	28.5 ± 10.3	25.4 ± 8.9	30.2 ± 8.7	29.1 ± 10.0	31.2 ± 11.4
P-value	-	< 0.001 ^d^	0.14	0.02 ^f^	< 0.001 ^d^	< 0.001 ^d^
**Serum ALP at study entry, IU/L**	171.3 ± 100.5	170.4 ± 87.4	176.1 ± 44.7	177.0 ± 50.8	162.0 ± 64.4	191.4 ± 153.5
P-value	-	0.94	0.86	0.89	0.64	0.46
**HBV DNA at study entry**						
Detectable	174 (58.8)	59 (57.3)	6 (37.5)	4 (66.7)	21 (55.3)	14 (66.7)
Undetectable	122 (41.2)	44 (42.7)	10 (62.5)	2 (33.3)	17 (44.7)	7 (33.3)
P-value	-	0.88	0.24	0.78	0.75	0.6

^a^ Abbreviations: AST, aspartate transaminase; ALT, alanine transaminase, ALP, alkaline phosphatase; HBV, hepatitis B virus; ULN, upper limit of normal.

^b^ Data are presented in No. (%) or Mean ± SD.

^c^ Estimated risk factors for elevation of serum ALT levels were compared with the cases of persistently normal ALT levels

^d^ Significant at P value < 0.001.

^e^ Significant at P value < 0.01.

^f^ Significant at P value < 0.05.

### 4.3. Natural Course of Inactive Carrier State

#### 4.3.1. Chronic Hepatitis B

**Figure 2.**
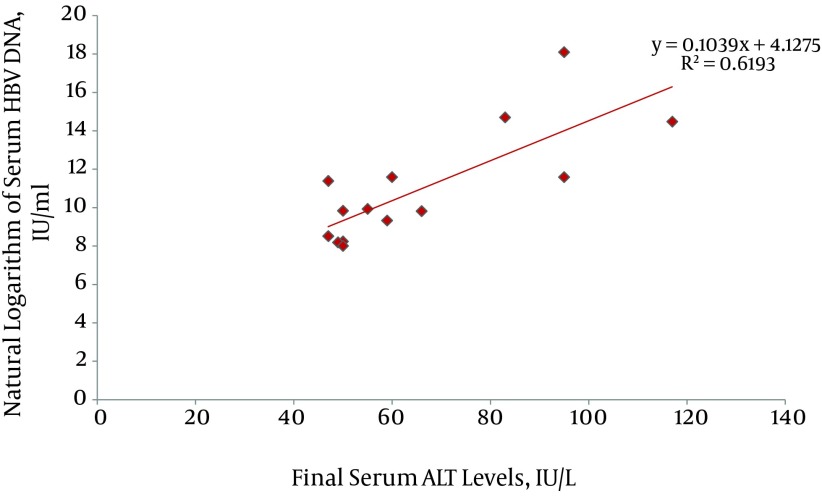
Association of Serum ALT Levels and Natural Logarithm of Serum HBV DNA in Patients with Chronic Hepatitis B

By the end of the study, 15 (3.76%) patients had developed HBeAg negative chronic hepatitis B (CHB); with an incidence rate of 0.42 per 100 person-years. Mean levels of ALT at the time of the CHB patients' diagnosis was 70.3 ± 27.4 IU/L; ranging from 47 to 131 IU/L. In addition, there was no significant difference in ALT levels between male and female patients (67.5 ± 29.6 IU/L and 81.3 ± 14.6 IU/L, respectively, P = 0.21). ALT levels at the time of diagnosis of CHB in 8, 2, 4 and 1 patient were within 1-1.5 × ULN, 1.5-2 × ULN, 2-3 × ULN, and 3-4 × ULN, respectively. Serum HBV DNA level at the time of diagnosis of CHB varied widely from 3.004 to > 7.2 × 107 IU/mL. Levels of ALT had a significant association with the natural logarithm of serum HBV DNA (P = 0.001, adjusted R square = 0.62) (Figure 2). Probable underlying risk factors for the progression of inactive HBV carrier state to HBeAg negative CHB were evaluated and described in Table 2. Interestingly, we found that patients with history of an episode of ALT elevation to > ULN and HBV DNA < 2 000 IU/mL (i.e. without an increase in HBV replication), in comparison to patients with persistently normal ALT levels, were at 8-fold higher risk for the development of HBeAg negative CHB (incidence rate 11.1% vs. 1.3%, RR = 8.3, 95% CI = 2.7–25.6, P < 0.001). Mean interval between the first episode of ALT elevation (with HBV DNA < 2 000 IU/mL) and the development of CHB was 58.1 ± 23.5 months; and this varied from 13 to 88 months.

**Table 2.** Estimated Risk Factors for Development of Chronic Hepatitis B in Inactive HBV Carrier State ^a^, ^b^, ^c^

Risk Factors	HBeAg Negative CHB (n = 15)	Inactive Carrier State (n=338)	P value
**Gender**			0.11
Male	12	192	
Female	3	148	
**Positive family Hx**	10 of 13 (76.9)	152 of 194 (78.4)	1.000
**Age at study entry, y**	31.3 ± 12.4	35.5 ± 11.6	0.17
**Age at study termination, y**	40.9 ± 13.1	44.4 ± 12.0	0.27
**Detectable serum HBV DNA at study entry**	8 of 15 (53.3)	203 of 340 (59.7)	0.71
**Mean HBV DNA at study entry, IU/mL**	831.7 ± 991.3	687.4 ± 887.6	0.818
**Serum AST at study entry, IU/L**	23.4 ± 8.5	24.5 ± 7.7	0.63
**Serum ALP at study entry, IU/L**	183.6 ± 181.2	170.6 ± 92.4	0.65
**Serum ALT at study entry, IU/L**	24.4 ± 11.8	23.8 ± 9.3	0.8
**Hx of **n**on-HBV related ALT elevation**	11 of 15 (73.3)	88 of 384 (22.9)	< 0.001 ^d^

^a^ Abbreviations: CHB, chronic hepatitis B; HBV, hepatitis B virus; AST, aspartate transaminase; ALT, alanine transaminase; ALP, alkaline phosphatase; Hx, history.

^b^ Data are presented in No. (%) or Mean ± SD.

^c^ Non-HBV related ALT elevation, elevated serum ALT Levels in absence of HBV replication (ie, HBV < 2 000 IU/mL)

^d^ significant at P value < 0.001.

#### 4.3.2. HBsAg Seroclearance

By the end of the study, negative results for serum HBsAg were observed in 48 patients. Finally, 43 (10.8%) patients maintained negative HBsAg to the end of the study and for a mean period of 24.6 ± 26.3 months; which revealed an annual incidence rate of 1.21% for seroclearance of HBsAg. In total, 12 patients also became seropositive for antibodies to hepatitis B surface antigens (HBsAb). No significant association was found between HBsAg seroclearance and gender, age, levels of AST, ALT, or HBV DNA, at study entry, or ALT fluctuations through the follow-ups (P > 0.05).

#### 4.3.3. Liver-Related Complications

Four patients (1 female, 3 males) developed cirrhosis by the end of the study. Two of the cases developed cirrhosis in the setting of CHB; while the remaining two patients were in an inactive carrier state. Moreover, one 37 year-old male patient with no history of ALT elevation developed HCC.

## 5. Discussion

In this long-term study, we followed up a cohort of 399 inactive HBsAg carrier patients with; positive serum HBsAg, negative HBeAg, low levels of HBV replication (serum HBV DNA < 2 000 IU/mL), and persistently normal levels of serum ALT (< 40 IU/L) at study entry. Through nine years of follow-up, we aimed to evaluate the incidence and prognostic values of serum ALT fluctuations on the development of chronic HBeAg negative hepatitis B, HBsAg seroclearance, and liver-related complications. Evaluation of the natural course of ALT fluctuations among patients with an inactive HBV carrier state is of value. According to the current guidelines (4, 5), ALT is the first-line modality to follow up these patients. Moreover, it has repeatedly been reported that higher levels of serum ALT in HBV chronic infection are associated with a higher risk for the incidence of hepatic histopathologic changes and cirrhosis (20, 21), and hepatocellular carcinoma (21-23). In our study, patients with a HBV inactive carrier state were at annual risk of 2.9% for elevation of ALT to > ULN measures. Through the nine years of follow-up, 25.8% of the patients experienced at least one episode of ALT elevation. However, in 63.1% of cases of ALT elevation, enzyme levels decreased to normal levels within six months. In addition, in 84.5% of the patients with ALT elevation, maximal serum levels of ALT were within 1-2 × ULN measures. And in 79.6% of the cases, only a single episode of ALT elevation was observed through the follow-ups. Finally, in 84.4% of the cases, elevations of ALT levels were not accompanied by increased HBV replication; ie, serum HBV PCR measures were < 2 000 IU/mL. The incidence of ALT elevation was significantly higher in patients of male gender, age less than 45 years, and higher levels of serum ALT at study entry.

Interpretation of fluctuations in serum ALT levels in HBV chronic carriers may be challenging, since elevations of ALT levels are a frequent finding in the general population, and may be associated with etiologies other than viral infections. Cross-sectional studies on different populations estimate that the prevalence of ALT > ULN could be within 8-30% of the general population (24-31). Irrespective of viral hepatitis, the risk factors for elevation of serum ALT levels include; male gender (27-29, 31), age (24), obesity (25-31), diabetes (25, 28, 30, 31), hyperlipidemia (24, 25, 28, 30, 31), moderate to heavy alcohol drinking (26, 29, 31), and drug-induced liver injury (DILI) (32, 33). Depending on the methodology and the population of the study, non-alcoholic fatty liver disease (NAFLD) is estimated to be the etiology in 24-77% of cases of serum ALT elevations (24, 28, 30, 34). In a study on HBeAg negative patients with elevated ALT levels and low level viremia, there were symptoms of hyperglycemia and hyperlipidemia detected in 24.5% and 49% of the patients, respectively. Liver biopsy assessments also showed the presence of NAFLD and CHB in 38.8% and 26.6% of the patients, respectively (35).

The findings of this study suggest that the majority of patients with a HBV inactive carrier state maintain persistently normal levels of serum ALT through long-term follow-ups. However, elevations of ALT levels to > ULN measures are common findings in these patients. ALT elevations are usually within 1-2 × ULN, and these are not associated with an increase in HBV replication, as enzyme levels often decrease to normal levels within six months and a recurrence of enzyme elevation in patients is rare. These findings are similar to the results of recently published data (36), which suggests a favorable natural course of serum ALT level fluctuations in HBV inactive carriers.

This study also suggests that the annual incidence of HBeAg-negative CHB in patients with HBV inactive carrier state is 0.42%, with a cumulative incidence of 3.76% through the study. This rate seems significantly lower than previously reported results in other population studies which estimated the annual incidence rate of 1-3% for developing this outcome (13). A possible explanation for this discrepancy may be due to the inclusion criteria of this study; i.e. treatment-naïve HBV inactive carrier state, no alcohol use, no concurrent medical disease, and normal abdominal ultrasonography and laboratory tests at study entry. One other possibility could be the difference in HBV genotypes affecting the population studies. It has previously been reported that the HBV genotype among our population study (Iran) is genotype D of the virus (37, 38), which is reported to be associated with a more favorable course of the disease (16, 18, 39).

In this study, patients with non-HBV related ALT elevation (i.e. ALT >ULN, HBV DNA < 2 000 IU/mL) and patients with HBeAg negative CHB (i.e. ALT > ULN, HBV DNA > 2 000 IU/mL) did not show any significant difference in maximal serum ALT levels at the time of enzyme elevation, or in the duration of the episode. This suggests that these two items may not predict the development of CHB in patients with a HBV inactive carrier state and elevated levels of serum ALT. However, in patients previously diagnosed as CHB, serum ALT levels showed a positive statistical correlation with the natural logarithm of serum HBV DNA measured by PCR assays. An interesting finding of this study was that 73% of CHB cases (11 out of 15) had a positive history for elevation of ALT levels while concurrent serum HBV DNA measures were < 2 000 IU/mL. This finding suggests that inactive HBV carriers, who have experienced an episode of non-HBV related ALT elevation, in comparison to persistently normal levels of ALT, are at an 8-fold higher risk for the development of HBeAg negative CHB in future follow-ups. The development of CHB occurred after a mean interval of 58.1 ± 23.5 months after the first episode of non-HBV related ALT elevation.

One explanation for this phenomenon might be the possibility that an active inflammatory process in the liver tissue may persist in the absence of HBV viremia and even after normalization of serum ALT levels. Another possibility is that, there may be an underlying non-HBV related pathogenesis in these patients, which increases their vulnerability to reactivation of the HBV infection into chronic hepatitis. However, for a more clear understanding of this phenomenon, further investigations may be required to confirm the findings of this study; and to probe for the expected pathogenesis of this process. 
