# Erratum to: Prognostic Values of Fluctuations in Serum Levels of Alanine Transaminase in Inactive Carrier State of HBV Infection [Published in Hepatitis Monthly. 2014 May; 14(5): e17537]

**DOI:** 10.5812/hepatmon.20685

**Published:** 2014-05-26

This erratum supplies corrected statements and proofs of an error made during publishing process.

Hereby, Publisher declares that the corrected email address of the corresponding author is: 

editor@hepatmon.com

Publishing Corporation.

